# Quantum microscopy of cells at the Heisenberg limit

**DOI:** 10.1038/s41467-023-38191-4

**Published:** 2023-04-28

**Authors:** Zhe He, Yide Zhang, Xin Tong, Lei Li, Lihong V. Wang

**Affiliations:** Caltech Optical Imaging Laboratory, Andrew and Peggy Cherng Department of Medical Engineering, Department of Electrical Engineering, California Institute of Technology, 1200 E. California Blvd., MC 138-78, Pasadena, CA 91125 USA

**Keywords:** Quantum optics, Imaging and sensing, Microscopy, Single photons and quantum effects

## Abstract

Entangled biphoton sources exhibit nonclassical characteristics and have been applied to imaging techniques such as ghost imaging, quantum holography, and quantum optical coherence tomography. The development of wide-field quantum imaging to date has been hindered by low spatial resolutions, speeds, and contrast-to-noise ratios (CNRs). Here, we present quantum microscopy by coincidence (QMC) with balanced pathlengths, which enables super-resolution imaging at the Heisenberg limit with substantially higher speeds and CNRs than existing wide-field quantum imaging methods. QMC benefits from a configuration with balanced pathlengths, where a pair of entangled photons traversing symmetric paths with balanced optical pathlengths in two arms behave like a single photon with half the wavelength, leading to a two-fold resolution improvement. Concurrently, QMC resists stray light up to 155 times stronger than classical signals. The low intensity and entanglement features of biphotons in QMC promise nondestructive bioimaging. QMC advances quantum imaging to the microscopic level with significant improvements in speed and CNR toward the bioimaging of cancer cells. We experimentally and theoretically prove that the configuration with balanced pathlengths illuminates an avenue for quantum-enhanced coincidence imaging at the Heisenberg limit.

## Abstract (web summary)

The authors present a method for super-resolution quantum microscopy at the Heisenberg limit by using pairs of entangled photons with balanced pathlengths. They improve the spatial resolution, imaging speed, and contrast-to-noise ratio in practice while providing a theoretical interpretation of the super-resolution feature.

## Introduction

Since the first demonstration of entangled photon sources, the biphoton state^1–3^ has found extensive applications in quantum computing^4^, quantum metrology^5,6^, and quantum information^7,8^. In particular, the nonclassical behavior of biphotons motivates the search for solutions that break classical limits, such as the uncertainty principle or the diffraction limit^9,10^. The diffraction pattern of biphotons has been demonstrated to be half as narrow as that of classical light^11–13^, indicating the capability of biphoton imaging to achieve super resolution beyond what is possible with classical light in diffraction-limited linear imaging^14^.

A variety of approaches have been proposed for quantum imaging using biphotons. Different nonlinear crystals, including β-barium borate (BBO)^15^ and periodically poled potassium titanyl phosphate (PPKTP)^16^, were used for generating entangled photon pairs utilizing the spontaneous parametric down-conversion (SPDC) effect^17–19^. In addition, different types of detectors were employed for biphoton detection. For example, single-photon avalanche diodes (SPADs) can provide direct coincidence measurements based on the arrival times of entangled photon pairs but do not have spatial resolution as they are single-pixel detectors. Though SPAD-array cameras add spatial resolution to single SPADs, they have a small number of pixels^15,20–22^. Electron multiplying charge-coupled devices (EMCCDs) provide a large number of resolvable pixels but are not capable of direct coincidence measurements due to the low frame rate^23–25^. Thus far, two methods have been developed and are frequently used to extract the coincidence counts from an EMCCD camera^23,24^. However, these methods typically require more than 2 × 10^6^ frames to generate a single coincidence image, which can take over 17 h, given the low frame rate. The development of wide-field quantum imaging, therefore, is hampered by the low acquisition rate. In comparison to classical wide-field imaging, quantum imaging benefits from stray light resistance^24,26^, enhancement of two-photon absorption^27,28^, and enhanced resolution as a result of quantum correlation^23,29^. Despite these advantages, EMCCD-based wide-field quantum imaging with spatial resolution as fine as 1.4 μm has never been reported due to the use of low-intensity light.

When quantum entanglement is applied for resolution beyond the classical limit^5^, *N* entangled photons may improve spatial resolution by *N* times, corresponding to the Heisenberg limit^30^. Using a biphoton NOON state in quantum lithography^1^ enhances resolution by two fold, and using an SPDC source also achieves the Heisenberg limit^11^. Both methods utilize co-propagating biphotons to enhance resolution by a factor of 2^30,31^. Moreover, recent studies indicate that resolution at the Heisenberg limit could be realized even without requiring both entangled photons to pass through the imaging object^23^.

Here, inspired by the wide-field imaging method introduced in refs. ^23,32^, we develop quantum microscopy by coincidence (abbreviated as QMC in our work) with balanced pathlengths using an EMCCD camera. Previous studies could not be used for practical microscopy for the following reasons. First, they did not have the option for high-resolution imaging due to the small numerical aperture (NA) of the imaging system. Second, they had a low imaging speed, requiring a large number of frames for coincidence measurements. In our technique, we improve the spatial resolution and speed by introducing a high-NA microscopy design and a more efficient algorithm. QMC relies on the nonclassical properties of biphotons for super-resolution microscopy with up to 5 times higher speeds, 2.6 times higher CNRs, and 10 times more resistance to stray light than existing wide-field quantum imaging techniques^23,24^. In contrast to the quantum imaging techniques at the standard quantum limit^1,29,33^, QMC improves resolution by a factor of 2 at the Heisenberg limit^23^. We demonstrate QMC for imaging cancer cells with a 1.4 μm resolution and a 100 × 50 μm^2^ field of view (FOV). The combination of the improved speed, enhanced CNR, more robust stray light resistance, super resolution, and low-intensity illumination empowers QMC toward bioimaging.

## Results

### Experimental setup

**Fig. 1:**
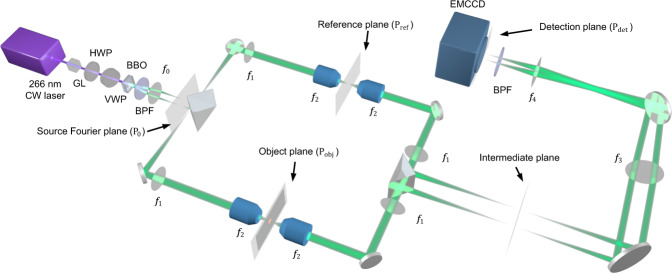
Experimental setup of QMC. CW continuous wave, GL Glan-Laser polarizer, HWP half-wave plate, VWP variable wave plate, BBO β-barium borate crystal, BPF 532 nm bandpass filter, PBS polarizing beam splitter, EMCCD electron multiplying charge-coupled device camera. *f*_0_ = 50 mm, *f*_1_ = 180 mm, *f*_2_ = 9 mm, *f*_3_ = 300 mm, and *f*_4_ = 200 mm. The source Fourier plane P_0_ is set at the Fourier plane of the BBO crystal.

The experimental setup of QMC is presented in Fig. 1 (see “Methods” for details). The first prism separates the signal and idler photons into two arms, and the optical pathlengths of the separated arms are balanced (see “Discussion”). The arm associated with the object is considered by itself the classical part for imaging, which can be regarded as a wide-field microscope. Therefore, an image acquired by this arm alone is deemed a classical image.

Despite being used to image distinct types of objects, the previous methods^23,24^ (Supplementary Fig. 1) and QMC are based on similar theories. However, the previous works demonstrated only macroscopic imaging because the two arms share the same lenses with a small NA and a large field of view (FOV). In comparison, QMC evenly splits the beam at the source Fourier plane into the signal and idler arms using a right-angle prism, which allows integrating high-NA objectives in each arm. As shown in Fig. 1, the two arms are built symmetrically to ensure balanced optical pathlengths and magnification ratios, which are the key conditions for super resolution at the Heisenberg limit (see “Discussion”).

### Estimation of coincidence

**Fig. 2:**
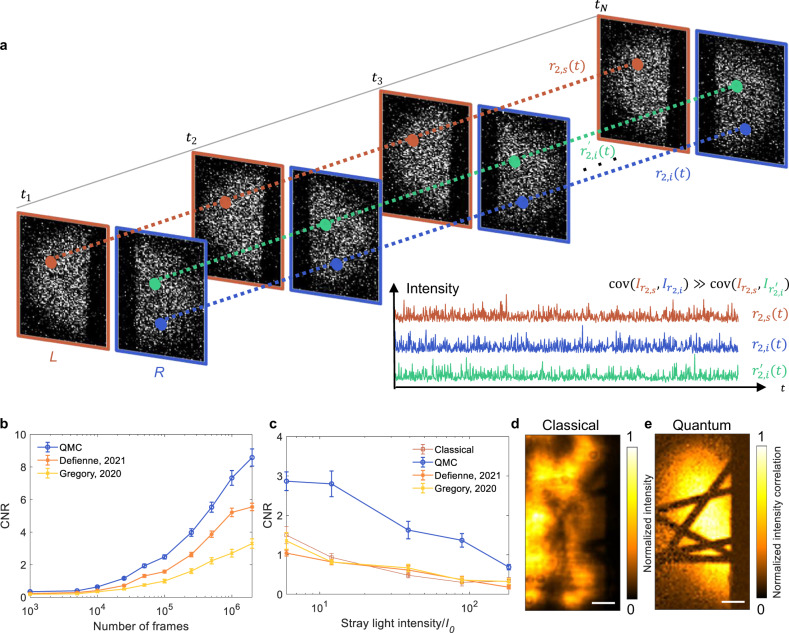
Coincidence measurement of QMC. **a** The coincidence measurement relies on the fact that the covariance between entangled photons in a sequence of frames is much larger than the covariance between two random photons. *L* and *R* refer to the left and right regions of the EMCCD, which are used to detect the signal and idler photons, respectively. **r**_2,*s*_ and **r**_2,*i*_ are symmetric positions on the detector for the signal and idler photons. **r**′_2,*i*_ is a random position in the right region and different from **r**_2,*i*_. Inset, intensities (*I*) at the three positions in different frames. **b** CNRs of QMC and the wide-field quantum imaging methods in refs. ^23,24^ using different numbers of frames. Data are plotted as means ± standard errors of the means (*n* = 10). **c** CNRs of QMC and the wide-field quantum imaging methods in refs. ^23,24^ with 10^5^ frames in the presence of stray light with different intensities. Data are plotted as means ± standard errors of the means (*n* = 10). The mean number of photons incident on the EMCCD is 0.49 per pixel per frame. Classical (**d**) and QMC (**e**) images of carbon fibers in the presence of stray light with an intensity of 8*I*_0_, acquired using 2 × 10^6^ frames. Scale bars, 20 μm.

As shown in Fig. 2a, we develop a covariance algorithm to efficiently estimate the coincidence intensity of signal and idler photons using an EMCCD camera. The signal and idler photons are detected by the left and right regions of the camera, respectively. The calibration for the EMCCD is shown in Supplementary Fig. 2. The total intensity ${I}^{L}({I}^{R})$ is related to the coincidence intensity of biphotons *I*_coin_ and the intensity of noise ${I}_{{{{{{\rm{noise}}}}}}}^{L}({I}_{{{{{{\rm{noise}}}}}}}^{R})$, where *L* and *R* represent the left and right regions, respectively. In QMC, we use the mean value of coincidence intensity $\overline{{I}_{{{{{{\rm{coin}}}}}}}}$ to estimate the intensity correlation ${G}_{{{{{{\rm{QMC}}}}}}}^{(2)}$. Studies have demonstrated that the distributions of entangled photon pairs in both regions are symmetric about a center point due to their momentum anticorrelation in the far field of the crystal^32^; therefore, the left and right images can be inversely registered pixel by pixel according to the symmetric center. The intensities of each pair of inversely registered pixels in the left and right images are given by

1 $${I}^{L}={I}_{{{{{{\rm{coin}}}}}}}+{I}_{{{{{{\rm{noise}}}}}}}^{L}.$$

2 $${I}^{R}={I}_{{{{{{\rm{coin}}}}}}}+{I}_{{{{{{\rm{noise}}}}}}}^{R}.$$

The covariance between ${I}^{L}$ and ${I}^{R}$ is defined by

3 $${{{{{\rm{cov}}}}}}({I}^{L},{I}^{R})=\frac{1}{N}{\sum }_{i}^{N}({I}_{i}^{L}-\bar{{I}^{L}})({I}_{i}^{R}-\bar{{I}^{R}}).$$

where *N* is the number of frames, and the subscript *i* refers to the frame index. Eq. (3) can be simplified to

4 $${{{{{\rm{cov}}}}}}({I}^{L},{I}^{R})=\overline{{I}_{{{{{{\rm{coin}}}}}}}^{2}}-{(\overline{{I}_{{{{{{\rm{coin}}}}}}}})}^{2}+\overline{{I}_{{{{{{\rm{noise}}}}}}}^{L}{I}_{{{{{{\rm{noise}}}}}}}^{R}}-\overline{{I}_{{{{{{\rm{noise}}}}}}}^{L}}\cdot \overline{{I}_{{{{{{\rm{noise}}}}}}}^{R}}.$$

while the first two terms represent the variance of the signal, the last two terms represent the covariance of the detection noise. Because the noise is primarily caused by the detector, which can be assumed to be uncorrelated between the left and right regions, the covariance of the detection noise approximately vanishes. In Supplementary Fig. 3, we demonstrate that $\overline{{I}_{{{{{{\rm{noise}}}}}}}^{L}{I}_{{{{{{\rm{noise}}}}}}}^{R}}-\overline{{I}_{{{{{{\rm{noise}}}}}}}^{L}}\cdot \overline{{I}_{{{{{{\rm{noise}}}}}}}^{R}}\ll \overline{{I}_{{{{{{\rm{coin}}}}}}}^{2}}-{\left(\overline{{I}_{{{{{{\rm{coin}}}}}}}}\right)}^{2}$. Further, as the coincidence intensity follows a Poisson distribution^17^, for which the variance equals the mean, we have^34^

5 $${{{{{\rm{cov}}}}}}({I}^{L},{I}^{R})=\overline{{I}_{{{{{{\rm{coin}}}}}}}^{2}}-{\left(\overline{{I}_{{{{{{\rm{coin}}}}}}}}\right)}^{2}=\overline{{I}_{{{{{{\rm{coin}}}}}}}}.$$

With enough frames, Eq. (5) directly estimates the expected value of the coincidence intensity, which is not directly provided by the existing algorithms^23,24,32^. Figure 2b compares QMC with the existing methods^23,24^ in terms of the CNR versus the number of frames. The CNR calculation workflow is shown in “Methods” and Supplementary Fig. 4. To achieve a CNR of 3, QMC requires 10^5^ frames (10 ms per frame), which is approximately 40% and 20% of the required frames in refs. ^23,24^, respectively. When 2 × 10^6^ frames are used, QMC outperforms the methods given in refs. ^23,24^ with 1.5 times and 2.6 times higher CNR, respectively (see Supplementary Fig. 4). As expected, CNR increases with the number of frames (Supplementary Fig. 5).

Resistance to stray light is a major advantage of quantum imaging. Eq. (5) indicates that the covariance algorithm suppresses uncorrelated noise, such as stray light. While the methods introduced in refs. ^23,24^ were reported to be effective in preventing stray light in images using over 2 × 10^6^ frames, their effectiveness is limited, especially when the frame number is less than 10^5^. Figure 2c shows the dependence of CNR on stray light intensity with 10^5^ frames. The data processing workflow for the stray light resistance in Fig. 2c is demonstrated in Supplementary Fig. 6. When the stray light intensity is ~12 times greater than the classical signal, the classical image is severely disrupted because the CNR falls below unity; the methods in refs. ^23,24^ cannot maintain the CNR either. Nonetheless, with 10^5^ frames, the CNR of QMC remains higher than unity even when the stray light is ~120 times stronger than the classical signal. Figures 2d, e display the classical and QMC images of carbon fibers in the presence of stray light that is 8 times stronger than the classical signal. Whereas the classical image is overwhelmed by the stray light (CNR = 0.92 ± 0.11), the QMC image eliminates the stray light nearly completely by extracting the coincidence intensity (CNR = 8.03 ± 1.22). In fact, with 2 × 10^6^ frames, QMC effectively suppresses stray light that is ~155 times stronger than the classical signal (Supplementary Fig. 7). The stray light resistance reaches its limit when the accidental coincidence caused by the stray light equals the true coincidence. The covariance algorithm proves to be the most effective at finding true coincidences of entangled photons and eliminating uncorrelated noise, providing the highest CNR under the same stray light intensity.

### Quantification of super resolution at the Heisenberg limit

**Fig. 3:**
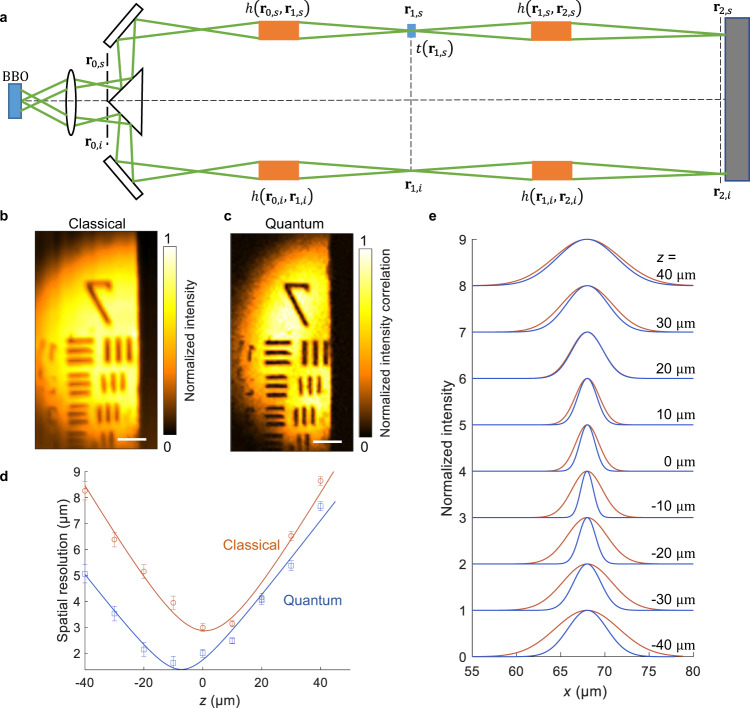
Spatial resolution of QMC. **a** Simple schematic of QMC. BBO, β-barium borate crystal. EMCCD, electron multiplying charge-coupled device camera. ${{{{{{\bf{r}}}}}}}_{0}$, ${{{{{{\bf{r}}}}}}}_{1}$, and ${{{{{{\bf{r}}}}}}}_{2}$ are the coordinates of the source Fourier plane, the object plane, and the detection plane, respectively. ${{{{{{\bf{r}}}}}}}_{0,s}$, ${{{{{{\bf{r}}}}}}}_{1,s}$, and ${{{{{{\bf{r}}}}}}}_{2,s}$ are the corresponding coordinates in the signal arm, and ${{{{{{\bf{r}}}}}}}_{0,i}$, ${{{{{{\bf{r}}}}}}}_{1,i}$, and ${{{{{{\bf{r}}}}}}}_{2,i}$ are the coordinates in the idler arm. $h\left({{{{{{\bf{r}}}}}}}_{0,s},{{{{{{\bf{r}}}}}}}_{1,s}\right)$ and $h\left({{{{{{\bf{r}}}}}}}_{1,s},{{{{{{\bf{r}}}}}}}_{2,s}\right)$ are the PSFs from ${{{{{{\bf{r}}}}}}}_{0,s}$ to ${{{{{{\bf{r}}}}}}}_{1,s}$ and from ${{{{{{\bf{r}}}}}}}_{1,s}$ to ${{{{{{\bf{r}}}}}}}_{2,s}$. $h\left({{{{{{\bf{r}}}}}}}_{0,i},{{{{{{\bf{r}}}}}}}_{1,i}\right)$ and $h\left({{{{{{\bf{r}}}}}}}_{1,i},{{{{{{\bf{r}}}}}}}_{2,i}\right)$ are the PSFs from ${{{{{{\bf{r}}}}}}}_{0,i}$ to ${{{{{{\bf{r}}}}}}}_{1,i}$ and from ${{{{{{\bf{r}}}}}}}_{1,i}$ to ${{{{{{\bf{r}}}}}}}_{2,i}$. $t$ is the amplitude transmission coefficient of the object. Classical (**b**) and QMC (**c**) images of group 7 (2.76 ~ 3.91 μm) of a USAF 1951 resolution target. Scale bars, 20 μm. All the images are normalized by their maximum and minimum intensities (see “Methods”). **d** Spatial resolution of classical imaging and QMC versus the axial *z* coordinate from the classical focal point. The highest spatial resolutions for classical imaging and QMC are 2.9 μm and 1.4 μm, respectively. Data are plotted as means ± standard errors of the means ($n=14$). **e** Normalized lateral LSFs of classical imaging and QMC versus the lateral coordinate at different *z* positions.

We next quantify the enhanced spatial resolution of QMC. Figure 3a shows a simplified schematic of QMC. Figure 3b demonstrates the classical image of group 7 of a US Air Force (USAF) resolution target that includes stripes of varying widths (from 2.76 to 3.91 μm), which approximate the highest resolution of our classical imaging setup. In Fig. 3c, the QMC image shows a higher resolution than the classical image. We evaluate the resolution enhancement by determining the full width at half maximum (FWHM) of the line spread functions (LSFs) near the focal point (see “Methods” for details). Figure 3d shows that the highest resolutions of classical imaging and QMC are 2.9 μm and 1.4 μm, respectively, indicating that QMC improves the spatial resolution of classical imaging by approximately a factor of 2. The LSFs at different axial *z* coordinates are shown in Fig. 3e.

QMC demonstrates a two-fold enhancement in spatial resolution over classical imaging due to the fact that the equivalent wavelength of the biphoton is half the wavelength of the SPDC photon. As shown in Fig. 3a, **r**_0,*s*_, **r**_1,*s*_, and **r**_2,*s*_ are the coordinates of the source Fourier plane, the object plane, and the detection plane in the signal arm, respectively. **r**_0,*i*_, **r**_1,*i*_, and **r**_2,*i*_ are the corresponding coordinates in the idler arm. The quantized field operators ${\hat{E}}_{s}^{\left(+\right)}$ and ${\hat{E}}_{i}^{\left(+\right)}$ for the signal and idler arms are shown in Supplementary Note 1. The intensity correlation between the signal and idler photons for a given pixel pair is given by the second-order correlation function:

6 $${G}_{{{{{{\rm{QMC}}}}}}}^{(2)}={\left |\left\langle 0 | {\hat{E}}_{s}^{\left(+\right)}{\hat{E}}_{i}^{\left(+\right)} | \xi \right\rangle \right |}^{2}.$$

where $\left|\xi \right\rangle$ is the wavefunction of a photon pair emitted from the source Fourier plane at **r**_0,*s*_ and **r**_0,*i*_:

7 $$\left | \xi \right\rangle={\sum }_{{{{{{{\bf{k}}}}}}}_{0,s}}A\left({{{{{{\bf{k}}}}}}}_{0,s}\right){e}^{-j{{{{{{\bf{k}}}}}}}_{0,s}\, \cdot \, {{{{{{\bf{r}}}}}}}_{0,s}}{e}^{-j{{{{{{\bf{k}}}}}}}_{0,i}\, \cdot \, {{{{{{\bf{r}}}}}}}_{0,i}}\left|1_{{{{{{{\bf{k}}}}}}}_{0,s}},{1}_{{{{{{{\bf{k}}}}}}}_{0,i}}\right\rangle .$$

**k**_0,*s*_ and **k**_0,*i*_ are the entangled wavevectors of the signal and idler photons from the source Fourier plane. Denoting **r**_*p*_ and **k**_*p*_ the position and wavevector of the pump light, Eq. (7) demonstrates a spatially entangled state with $\left({{{{{{\bf{r}}}}}}}_{0,s}{{{{{\boldsymbol{+}}}}}}{{{{{{\bf{r}}}}}}}_{0,i}\right)/2{{{{{\boldsymbol{=}}}}}}{{{{{{\bf{r}}}}}}}_{p}$ and ${{{{{{\bf{k}}}}}}}_{0,s}{{{{{\boldsymbol{+}}}}}}{{{{{{\bf{k}}}}}}}_{0,i}{{{{{\boldsymbol{=}}}}}}{{{{{{\bf{k}}}}}}}_{p}$. $A\left({{{{{{\bf{k}}}}}}}_{0,s}\right)$ denotes the probability amplitude of the state $|1_{{{{{{{\rm{k}}}}}}}_{0,s}},{1}_{{{{{{{\rm{k}}}}}}}_{0,i}}\rangle$. The momentum correlation width of the entangled photons is demonstrated in Supplementary Fig. 8.

As derived in Supplementary Note 1, the QMC image can be described by ${G}_{{{{{{\rm{QMC}}}}}}}^{\left(2\right)}:$

8 $${G}_{{{{{{\rm{QMC}}}}}}}^{\left(2\right)}\left({{{{{\boldsymbol{\rho }}}}}}\right)={{{{{{\rm{|}}}}}}t\left({{{{{\boldsymbol{\rho }}}}}}\right){{{{{\rm{|}}}}}}}^{2}{\varGamma}_{{{{{{\rm{QMC}}}}}}}\left(\frac{\lambda }{2}{{{{{\rm{;}}}}}}{{{{{\boldsymbol{\rho}}}}}} \right){\left | h\left(\frac{\lambda }{2}{{{{{\rm{;}}}}}}{{{{{\boldsymbol{\rho }}}}}},{M}{{{{{{\boldsymbol{\rho }}}}}}}\right)\right | }^{2}.$$

where *t* (**ρ**) is the amplitude transmission coefficient of the object, and **ρ** is the 2D coordinates on the object plane. ${\varGamma }_{{{{{{\rm{QMC}}}}}}}\left(\frac{\lambda }{2};{{{{{\boldsymbol{\rho }}}}}}\right)$ is the distribution of squared intensity with a wavelength of *λ*/2 on the object plane. *h* (*λ*;**ρ**,*M***ρ**) is the point spread function (PSF) from **ρ** on the object plane (P_obj_) to *M***ρ** on the detection plane (P_det_) for the light with a wavelength of *λ*/2. *M* is the magnification ratio from the object to the detector. Similarly, the classical counterpart is given by

9 $${G}_{{{{{{\rm{CI}}}}}}}^{(1)}\left({{{{{\boldsymbol{\rho }}}}}}\right)={\left|t\left({{{{{\boldsymbol{\rho }}}}}}\right)\right|}^{2}{\gamma }_{{{{{{\rm{CI}}}}}}}\left(\lambda {{{{{\rm{;}}}}}}{{{{{\boldsymbol{\rho }}}}}}\right){\left|h\left(\lambda {{{{{\rm{;}}}}}}{{{{{\boldsymbol{\rho }}}}}},M{{{{{\boldsymbol{\rho }}}}}}\right)\right|}^{2}.$$

where ${\gamma }_{{{{{{\rm{CI}}}}}}}\left(\lambda ;{{{{{\boldsymbol{\rho }}}}}}\right)$ is the intensity distribution of wide-field illumination with a wavelength of *λ* on the object plane. $h\left(\lambda ;{{{{{\boldsymbol{\rho }}}}}},M{{{{{\boldsymbol{\rho }}}}}}\right)$ denotes the point spread function (PSF) from **ρ** on the object plane (P_obj_) to *M***ρ** on the detection plane (P_det_) for the light with a wavelength of *λ*.

In contrast to linear classical imaging, QMC is based on a pure quantum effect to achieve the Heisenberg limit. Unlike the quantum super-resolution methods requiring both signal and idler photons to pass through the object^30^, the idler photons in our experiment do not traverse the object. Indicated by Eqs. (S5) and (S6), while calculating the QMC image, the point spread functions $h\left({{{{{{\bf{r}}}}}}}_{1,s},{{{{{{\bf{r}}}}}}}_{2,s}\right)$ and $h\left({{{{{{\bf{r}}}}}}}_{1,i},{{{{{{\bf{r}}}}}}}_{2,i}\right)$ in Fig. 3a are multiplied for each photon pair instead of being multiplied classically as $h\cdot h={h}^{2}$. This concept has been theoretically proven for quantum imaging based on a simplified model^35^, which, however, cannot be applied to the complex setup in this work (see Fig. 3a). For example, as shown in Fig. 3b, c, the spatial distributions of the wide-field illumination for the classical imaging and QMC are different, which was not considered in ref. ^35^ but can be explained by different wide-field illumination functions ${\varGamma }_{{{{{{\rm{QMC}}}}}}}\left(\lambda /2;{{{{{\boldsymbol{\rho }}}}}}\right)$ and ${\gamma }_{{{{{{\rm{CI}}}}}}}\left(\lambda ;{{{{{\boldsymbol{\rho }}}}}}\right)$ in Eqs. (8, 9).

The classical resolution in our experiment is limited by the effective NA of the objectives, which may be lower than the nominal NA of the objectives (NA = 0.4) due to underfilling.

### Imaging cells by QMC

**Fig. 4:**
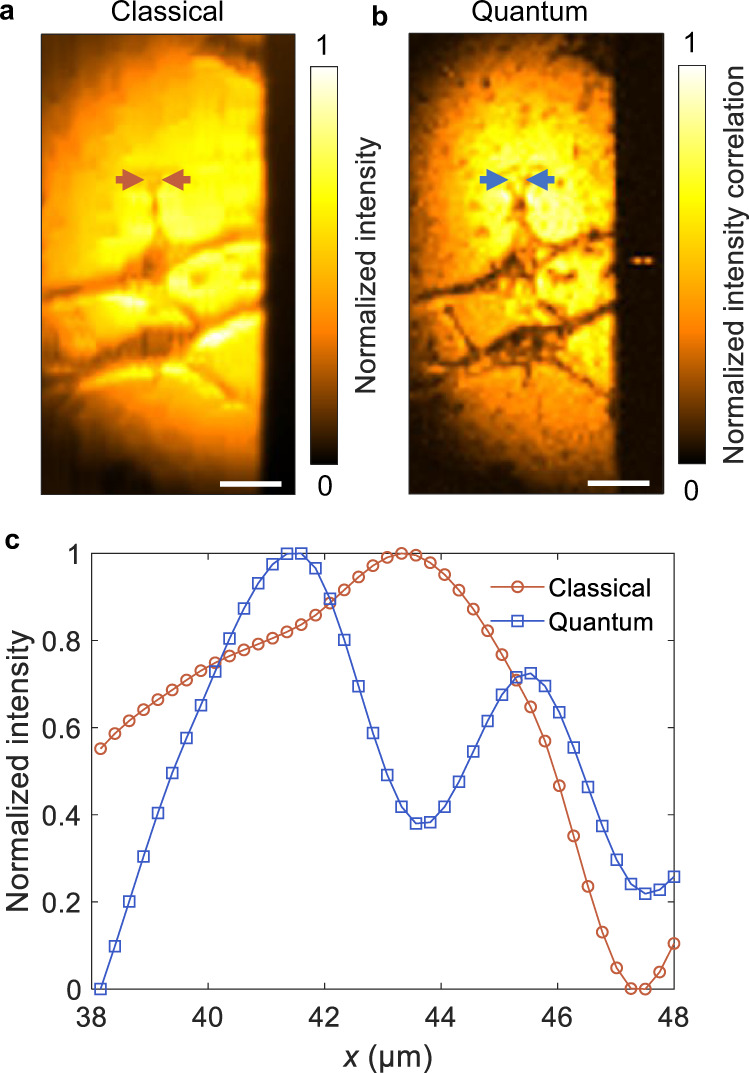
Imaging of cancer cells with QMC. Classical (**a**) and QMC (**b**) images of two HeLa cells. Scale bars, 20 μm. **c** Normalized classical and QMC intensities between the arrows in (**a**) and (**b**).

In Fig. 4, we demonstrate classical (Fig. 4a) and QMC (Fig. 4b) imaging of cancer cells. Figure 4c shows the normalized intensities between the arrows in Fig. 4a, b. QMC clearly distinguishes the cell structures that cannot be resolved in its classical counterpart. Note that the lumpy features in both images are due to imperfect sample preparation. Whereas the images in Fig. 4 were averaged over 2 × 10^6^ frames (10 ms per frame) to achieve a high CNR, the images of the cells shown in Supplementary Fig. 9 were acquired using fewer (10^5^) frames.

## Discussion

The balanced pathlengths require symmetry in the optical paths of the signal and idler photons from the source Fourier plane to the detection planes, such that the paired photons are correlated in positions and momentums concurrently, and the phases of the paired photons can be combined. This requirement, however, cannot be satisfied through classical sources because two unentangled photons can only be correlated in either position or momentum in accordance with the uncertainty principle^36^. As a consequence of the path symmetry, all entangled photon pairs should appear at positions symmetric about the same center within the source Fourier plane, the object plane, and the detection plane. We mirrored the signal arm setup onto the idler arm to maintain the path symmetry as precisely as possible. In particular, the photon pairs on the symmetric positions on the source Fourier plane propagate symmetrically due to the SPDC phase matching, and they propagate through the identical pairs of free-space 4*f* systems to reach the object plane and the reference plane, respectively. The signal photon on the object plane can be scattered by the object, leading to different wavevectors between the signal and idler photons. In Fig. 3a, in the paraxial approximation, photons traversing the same position on the object plane would arrive at the same position on the detection plane, indicating identical optical pathlengths according to Fermat’s principle. Though the scattering effect appears to disrupt the path symmetry, the pathlength symmetry is maintained because the conjugation between the object and detection planes balances the optical pathlengths of a scattered signal photon and the related idler photon. Therefore, we can utilize the configuration of the balanced pathlengths to describe the biphoton propagation from the source Fourier plane to the detection plane (see Eq. (S5)).

We have attempted to provide the most practical setup and algorithm for quantum bioimaging with a spatial resolution down to 1.4 μm. However, the current implementation of QMC is not intended to compete with state-of-the-art classical microscopy techniques in terms of CNRs because of the low SPDC efficiency of the BBO crystal. For example, to achieve a CNR of 3, QMC requires the acquisition of about 10^5^ frames over 17 min, whereas classical imaging may only need a single frame captured in less than a second. With more powerful quantum sources in the future^37^, QMC could demonstrate quantum advantages over state-of-the-art classical imaging. Furthermore, compared with classical methods, such as SHG microscopy^38^, that reject background noise through spectral filtering, QMC eliminates both temporally and spatially uncorrelated background noise through coincidence detection.

In conclusion, we have demonstrated quantum microscopy of cancer cells at the Heisenberg limit. QMC is advantageous over existing wide-field quantum imaging methods due to the 1.4 μm resolution, up to 5 times higher speed, 2.6 times higher CNR, and 10 times more robustness to stray light. With low-intensity illumination, we have demonstrated that QMC is suitable for nondestructive bioimaging at a cellular level, revealing details that cannot be resolved by its classical counterpart. Finally, while the resolution of classical imaging can be improved in various ways^39,40^, the configuration used in QMC can further improve the resolution by halving the wavelength, thus pushing the boundary of classical super-resolution imaging techniques with quantum enhancement.

## Methods

### Experimental setup

In the QMC system, a β-barium borate (BBO) crystal (5 × 5 × 0.5 mm^3^, PABBO5050-266(I)-HA3, Newlight Photonics) was cut for type-I SPDC at 266 nm wavelength. The pump was a 266 nm continuous-wave laser (FQCW266-10-C, CryLaS) with an output power of 10 mW. A UV-coated Glan-Laser polarizer (GLB10-UV, Thorlabs) and a half-wave plate (WPH05M-266, Thorlabs) were used to adjust the polarization angle of the pump laser beam. For imaging, the pump beam was adjusted to be vertically polarized. The pump laser beam then passed through the BBO crystal and generated a ring of SPDC photons with a half-opening angle of 3°. A bandpass filter with a center wavelength of 532 nm and a bandwidth of 2 nm (64-252, Edmund Optics) was used to block the pump beam. The generated SPDC photon pairs propagated through an *f*_0_ = 50 mm lens to the Fourier plane, i.e., the source Fourier plane (P_0_), and were spatially separated using a knife-edge right-angle prism mirror (MRAK25-P01, Thorlabs). The separated signal and idler photons propagated to the object plane (P_obj_) and the reference plane (P_ref_), respectively, by two identical 4*f* imaging systems comprising of an *f*_1_ = 180 mm lens and an *f*_2_ = 9 mm objective (LI-20X, 0.4 NA, Newport). The sample was placed on the object plane. The object plane, the reference planes, and the intermediate plane were conjugate through the other two identical 4*f* imaging systems, which consist of an identical set of *f*_2_ = 9 mm objectives and *f*_1_ = 180 mm lenses and another right-angle prism mirror. Each objective was followed by an HWP mounted on a motorized precision rotation mount (PRM1Z8, Thorlabs). The intermediate plane and the detection plane (P_det_) of an EMCCD camera (iXon Ultra 888, Andor) were conjugated through a 4*f* system consisting of *f*_3_ = 300 mm and *f*_4_ = 200 mm lenses. Another BPF was placed in front of the EMCCD camera to block unwanted stray light. The EMCCD was operated at −65 °C, with a horizontal pixel shift readout rate of 10 MHz, a vertical pixel shift speed of 1.13 µs, and an electron multiplier (EM) gain of 1000. The whole setup was covered by a light-shielding box.

### Sample preparation

A 2” × 2” (5.08 × 5.08 cm) positive 1951 USAF resolution target (58-198, Edmund Optics) was used to quantify the spatial resolution and DOF of our system. The carbon fiber sample was prepared by randomly distributing carbon fibers with a diameter of 6 µm on top of a glass slide. The fibers were mixed with UV-curing optical adhesive (NOA61, Thorlabs) and sealed with a cover glass. The optical adhesive was then cured by illumination of UV light from a light-emitting diode (LED). HeLa cells were placed on sterile glass slides and cultured in DMEM supplemented with 10% fetal bovine serum and a penicillin-streptomycin mixture (all from Invitrogen/Life Technologies) at 37 °C in a 5% CO_2_ air atmosphere. When cells were 70% confluent on the glass slides, we fixed them with an ice-cold mixture of ethanol and methanol (1:1 volume ratio). The glass slides placed in a 10-cm petri-dish were covered by the organic solvents and then incubated in a freezer (−20 °C) for 5–7 min. The organic solvents preserved the cells by removing lipids, dehydrating tissue, and denaturing and precipitating the proteins in the cells. After fixation, the glass slides were gently rinsed with phosphate-buffered saline to remove any fixation agent.

### Data acquisition and processing

A custom-written LabVIEW (National Instruments) program utilizing the library from the Andor software development kit (SDK) was used to control the EMCCD for data acquisition. The imaging data were saved as 16-bit Flexible Image Transport System (FITS) files with each file containing 1000 frames. The FITS files were imported into MATLAB (MathWorks) and processed with custom-written scripts. The EMCCD frames were extracted from the files and were used to calculate the coincidence intensity using our QMC algorithm. The reconstructed images were then interpolated based on a cubic spline using not-a-knot end conditions for better visualization. The maximum pixel number of the EMCCD camera is 1024 × 1024. We utilized an area of 100 × 50 pixels after binning of 2.

### Image normalization

Denoting *I* as the image intensity, the normalized intensity is calculated by

10 $${I}_{{{{{{\rm{norm}}}}}}}=\frac{I-{I}_{{{\min }}}}{{I}_{{{\max }}}-{I}_{{{\min }}}}.$$

where *I*_max_ and *I*_min_ are the maximum and minimum values of *I*.

### Contrast-to-noise ratio estimation

Denoting *I*_1_ and *I*_2_ as the intensities of the object of interest and the background, respectively, the contrast-to-noise ratio (CNR) is calculated by

11 $${{{{{\rm{CNR}}}}}}=\frac{\left | {\bar{I}}_{1}-{\bar{I}}_{2}\right | }{\sqrt{{\sigma }_{1}^{2}+{\sigma }_{2}^{2}}}.$$

where ${\bar{I}}_{1}$ and ${\bar{I}}_{2}$ are the mean values; *σ*_1_ and *σ*_2_ are the standard deviations of *I*_1_ and *I*_2_.

### Measurements of resolution and depth of field

To measure the resolution of our system, the line profile perpendicular to an edge in the USAF resolution target was extracted and fitted to an edge spread function (ESF) centered at *x*_0_, i.e., ${{{{{\rm{ESF}}}}}}\left(x\right)=a{{{{{\rm{erf}}}}}}\left((x-{x}_{0})/w\right)+b$, where *a* and *b* are coefficients, and *w* is the radius of the beam. A Gaussian line spread function (LSF) was obtained by taking the derivative of the ESF, i.e., ${{{{{\rm{LSF}}}}}}\left(x\right)=d{{{{{\rm{ESF}}}}}}(x)/{dx}=2a\, {{\exp }}(-{\left(x-{x}_{0}\right)}^{2}/{w}^{2})/\left(w\sqrt{\pi }\right)$. The resolution was estimated by the FWHM of the LSF, i.e., ${{{{{\mathcal{R}}}}}}=2\sqrt{{{{{{\rm{ln}}}}}}2}w$.

### Imaging with stray light

A 532 nm continuous-wave laser (MLL-III-532, CNI) was used to introduce stray light to the detection plane. Transmitting through a ground glass diffuser (DG20-1500, Thorlabs), the 532 nm laser created speckle patterns on the detection plane, leading to significantly reduced CNR in the raw EMCCD images. To evaluate how robust the classical imaging and QMC were against the stray light, we acquired images under different stray light intensities and calculated their CNRs.

### Statistics and reproducibility

Statistical analysis was performed using MATLAB (R2021a). Data are presented as means ±  standard errors of the means in all figure parts in which error bars are shown. No statistical method was used to predetermine sample sizes. We determined sample sizes based on our preliminary studies and on the criteria in the field to experimentally demonstrate the imaging system. All experiments except for that shown in Fig. 4 were replicated at least twice. All attempts at replication were successful. Cell imaging in Fig. 4 with 2 × 10^6^ frames was not replicated because we repeated the measurement under the same condition with 10^5^ frames (Supplementary Fig. 9). Besides, we have repeated experiments on other samples under the same condition with 2 × 10^6^ frames.

### Reporting summary

Further information on research design is available in the Nature Portfolio Reporting Summary linked to this article.

## Supplementary information

Supplementary information

Reporting Summary

## Data Availability

Imaging data for the cell images generated in Fig. 4 are available in the Github online at http://github.com/ZheHE2022/Quantum-Microscopy-of-Cells-at-the-Heisenberg-Limit. All data used in this study are available from the corresponding author upon reasonable request.

## Code availability

The code for the covariance algorithm is provided in the Supplementary Software and Github online at http://github.com/ZheHE2022/Quantum-Microscopy-of-Cells-at-the-Heisenberg-Limit. All custom codes used in this study are available from the corresponding author upon reasonable request.

## References

[1] Moreau P-A, Toninelli E, Gregory T, Padgett MJ (2019). Imaging with quantum states of light. Nat. Rev. Phys..

[2] Shih Y (2003). Entangled biphoton source—property and preparation. Rep. Prog. Phys..

[3] Horodecki R, Horodecki P, Horodecki M, Horodecki K (2009). Quantum entanglement. Rev. Mod. Phys..

[4] O’Brien JL (2007). Optical quantum computing. Science.

[5] Giovannetti V, Lloyd S, Maccone L (2011). Advances in quantum metrology. Nat. Photonics.

[6] Taylor MA, Bowen WP (2016). Quantum metrology and its application in biology. Phys. Rep..

[7] Flamini F, Spagnolo N, Sciarrino F (2018). Photonic quantum information processing: a review. Rep. Prog. Phys..

[8] Magaña-Loaiza OS, Boyd RW (2019). Quantum imaging and information. Rep. Prog. Phys..

[9] Kolobov MI, Fabre C (2000). Quantum limits on optical resolution. Phys. Rev. Lett..

[10] Busch P, Shilladay C (2006). Complementarity and uncertainty in Mach–Zehnder interferometry and beyond. Phys. Rep..

[11] D’Angelo M, Chekhova MV, Shih Y (2001). Two-photon diffraction and quantum lithography. Phys. Rev. Lett..

[12] Mitchell MW, Lundeen JS, Steinberg AM (2004). Super-resolving phase measurements with a multiphoton entangled state. Nature.

[13] Afek I, Ambar O, Silberberg Y (2010). High-NOON states by mixing quantum and classical light. Science.

[14] Bennink RS, Bentley SJ, Boyd RW, Howell JC (2004). Quantum and classical coincidence imaging. Phys. Rev. Lett..

[15] Ndagano B (2022). Quantum microscopy based on Hong–Ou–Mandel interference. Nat. Photonics.

[16] Lemos GB (2014). Quantum imaging with undetected photons. Nature.

[17] Couteau C (2018). Spontaneous parametric down-conversion. Contemp. Phys..

[18] Zhang H (2011). Preparation and storage of frequency-uncorrelated entangled photons from cavity-enhanced spontaneous parametric downconversion. Nat. Photonics.

[19] Wagenknecht C (2010). Experimental demonstration of a heralded entanglement source. Nat. Photonics.

[20] Camphausen R (2021). A quantum-enhanced wide-field phase imager. Sci. Adv..

[21] Defienne H (2022). Pixel super-resolution with spatially entangled photons. Nat. Commun..

[22] Ndagano B (2020). Imaging and certifying high-dimensional entanglement with a single-photon avalanche diode camera. NPJ Quantum Inf..

[23] Defienne H, Ndagano B, Lyons A, Faccio D (2021). Polarization entanglement-enabled quantum holography. Nat. Phys..

[24] Gregory T, Moreau P-A, Toninelli E, Padgett MJ (2020). Imaging through noise with quantum illumination. Sci. Adv..

[25] Devaux F, Mosset A, Bassignot F, Lantz E (2019). Quantum holography with biphotons of high Schmidt number. Phys. Rev. A.

[26] Gilaberte Basset M (2019). Perspectives for applications of quantum imaging. Laser Photonics Rev..

[27] Varnavski O, Goodson T (2020). Two-photon fluorescence microscopy at extremely low excitation intensity: the power of quantum correlations. J. Am. Chem. Soc..

[28] Schlawin F, Dorfman KE, Mukamel S (2018). Entangled two-photon absorption spectroscopy. Acc. Chem. Res..

[29] Toninelli E (2019). Resolution-enhanced quantum imaging by centroid estimation of biphotons. Optica.

[30] Unternährer M, Bessire B, Gasparini L, Perenzoni M, Stefanov A (2018). Super-resolution quantum imaging at the Heisenberg limit. Optica.

[31] Xu D-Q (2015). Experimental observation of sub-Rayleigh quantum imaging with a two-photon entangled source. Appl. Phys. Lett..

[32] Defienne H, Reichert M, Fleischer JW (2018). General model of photon-pair detection with an image sensor. Phys. Rev. Lett..

[33] Meda A (2017). Photon-number correlation for quantum enhanced imaging and sensing. J. Opt..

[34] Sanders BC (2012). Review of entangled coherent states. J. Phys. A: Math. Theor..

[35] Santos IF, Sagioro MA, Monken CH, Pádua S (2003). Resolution and apodization in images generated by twin photons. Phys. Rev. A.

[36] Paneru D, Cohen E, Fickler R, Boyd RW, Karimi E (2020). Entanglement: quantum or classical?. Rep. Prog. Phys..

[37] Li L (2020). Metalens-array–based high-dimensional and multiphoton quantum source. Science.

[38] Campagnola PJ, Dong C-Y (2011). Second harmonic generation microscopy: principles and applications to disease diagnosis. Laser Photonics Rev..

[39] Saxena M, Eluru G, Gorthi SS (2015). Structured illumination microscopy. Adv. Opt. Photonics.

[40] Schermelleh L (2019). Super-resolution microscopy demystified. Nat. Cell Biol..

