# Supplementary material for: Quantum microscopy of cells at the Heisenberg limit
Source: Nat Commun. 2023 Apr 28;14:2441. doi: 10.1038/s41467-023-38191-4 (PMC10147633; doi:10.1038/s41467-023-38191-4)
Supplement: Supplementary file 1 — Supplementary information [file 41467_2023_38191_MOESM1_ESM.pdf]

Supplementary Information

Quantum Microscopy of Cells at the Heisenberg Limit

Zhe He†, Yide Zhang†, Xin Tong†, Lei Li, and Lihong V. Wang*

Caltech Optical Imaging Laboratory, Andrew and Peggy Cherng Department of Medical Engineering, Department of Electrical Engineering, California Institute of Technology, 1200 E. California Blvd., MC 138-78, Pasadena, CA 91125, USA

† These authors contributed equally.

* Correspondence should be addressed to L.V.W. (LVW@caltech.edu).

Supplementary Note 1

For the signal (idler) arm, let $\hat{O}$ and $\hat{O}'$ be the operators for the $4f$ systems before and after the object (reference) plane, respectively. $\hat{E}_s^{(+)}$ and $\hat{E}_i^{(+)}$ are the quantized field operators for the signal and idler arms, respectively, which are given in the absence of an object by

$$\hat{E}_s^{(+)}(\mathbf{r}_{2,s},\mathbf{r}_{1,s},\mathbf{r}_{0,s}) = E_0(\mathbf{r}_{0,s})\hat{e}|0_{\mathbf{r}_{2,s}}\rangle\langle 1_{\mathbf{r}_{2,s}}|\hat{O}'\,|1_{\mathbf{r}_{1,s}}\rangle\langle 1_{\mathbf{r}_{1,s}}|\hat{O}, \tag{S1}$$

$$\hat{E}_i^{(+)}(\mathbf{r}_{2,i},\mathbf{r}_{1,i},\mathbf{r}_{0,i}) = E_0(\mathbf{r}_{0,i})\hat{e}|0_{\mathbf{r}_{2,i}}\rangle\langle 1_{\mathbf{r}_{2,i}}|\hat{O}'\,|1_{\mathbf{r}_{1,i}}\rangle\langle 1_{\mathbf{r}_{1,i}}|\hat{O}\,, \tag{S2}$$

where $E_0(\mathbf{r}_{0,s})$ and $E_0(\mathbf{r}_{0,i})$ are the amplitudes of the electric field of the SPDC photons emitted from the source Fourier plane at $\mathbf{r}_{0,s}$ and $\mathbf{r}_{0,i}$. $\hat{e}$ is the unit vector for polarization. In the presence of an object, we expand both operators over $\mathbf{k}_{0,s},\mathbf{k}_{1,s},\mathbf{k}'_{1,s}$, and $\mathbf{k}_{2,s}$ and modify $\hat{E}_s^{(+)}$ with $t_0(\mathbf{r}_{1,s},\mathbf{k}_{1,s})$, the amplitude transmission coefficient of the object for the photon with the wavevector $\mathbf{k}_{1,s}$:

$$\begin{aligned}
&\hat{E}_s^{(+)}(\mathbf{r}_{2,s},\mathbf{r}_{1,s},\mathbf{r}_{0,s})\\
&= \sum_{\mathbf{k}_{0,s},\mathbf{k}_{1,s},\mathbf{k}'_{1,s},\mathbf{k}_{2,s}} E_0(\mathbf{r}_{0,s})\hat{e}|0_{\mathbf{r}_{2,s}}\rangle\langle 1_{\mathbf{r}_{2,s}}||1_{\mathbf{k}_{2,s}}\rangle\langle 1_{\mathbf{k}_{2,s}}|\hat{O}'\,\left|1_{\mathbf{k}'_{1,s}}\right\rangle\left\langle 1_{\mathbf{k}'_{1,s}}\right||1_{\mathbf{r}_{1,s}}\rangle t_0(\mathbf{r}_{1,s},\mathbf{k}_{1,s})\\
&\qquad\times\langle 1_{\mathbf{r}_{1,s}}||1_{\mathbf{k}_{1,s}}\rangle\langle 1_{\mathbf{k}_{1,s}}|\hat{O}|1_{\mathbf{k}_{0,s}}\rangle\langle 1_{\mathbf{k}_{0,s}}|\\
&= \sum_{\mathbf{k}_{0,s},\mathbf{k}_{1,s},\mathbf{k}'_{1,s},\mathbf{k}_{2,s}} E_0(\mathbf{r}_{0,s})\hat{e}|0_{\mathbf{r}_{2,s}}\rangle\langle 1_{\mathbf{r}_{2,s}}||1_{\mathbf{k}_{2,s}}\rangle\hat{O}'_{\mathbf{k}_{2,s},\mathbf{k}'_{1,s}}\left\langle 1_{\mathbf{k}'_{1,s}}\right||1_{\mathbf{r}_{1,s}}\rangle\, t_0(\mathbf{r}_{1,s},\mathbf{k}_{1,s})\\
&\qquad\times\langle 1_{\mathbf{r}_{1,s}}||1_{\mathbf{k}_{1,s}}\rangle\hat{O}_{\mathbf{k}_{1,s},\mathbf{k}_{0,s}}\langle 1_{\mathbf{k}_{0,s}}|\\
&= \sum_{\mathbf{k}_{0,s},\mathbf{k}_{1,s},\mathbf{k}'_{1,s},\mathbf{k}_{2,s}} E_0(\mathbf{r}_{0,s})\hat{e}|0_{\mathbf{r}_{2,s}}\rangle h(\mathbf{r}_{2,s},\mathbf{k}_{2,s};\mathbf{r}_{1,s},\mathbf{k}'_{1,s})t_0(\mathbf{r}_{1,s},\mathbf{k}_{1,s})\langle 1_{\mathbf{r}_{1,s}}||1_{\mathbf{k}_{1,s}}\rangle\hat{O}_{\mathbf{k}_{1,s},\mathbf{k}_{0,s}}\langle 1_{\mathbf{k}_{0,s}}|,
\end{aligned} \tag{S3}$$

$$\begin{aligned}
&\hat{E}_i^{(+)}(\mathbf{r}_{2,i},\mathbf{r}_{1,i},\mathbf{r}_{0,i})\\
&= \sum_{\mathbf{k}_{0,i},\mathbf{k}_{1,i},\mathbf{k}'_{1,i},\mathbf{k}_{2,i}} E_0(\mathbf{r}_{0,i})\hat{e}|0_{\mathbf{r}_{2,i}}\rangle\langle 1_{\mathbf{r}_{2,i}}||1_{\mathbf{k}_{2,i}}\rangle\langle 1_{\mathbf{k}_{2,i}}|\hat{O}'\,\left|1_{\mathbf{k}'_{1,i}}\right\rangle\left\langle 1_{\mathbf{k}'_{1,i}}\right||1_{\mathbf{r}_{1,i}}\rangle\\
&\qquad\times\langle 1_{\mathbf{r}_{1,i}}||1_{\mathbf{k}_{1,i}}\rangle\langle 1_{\mathbf{k}_{1,i}}|\hat{O}|1_{\mathbf{k}_{0,i}}\rangle\langle 1_{\mathbf{k}_{0,i}}|\\
&= \sum_{\mathbf{k}_{0,i},\mathbf{k}_{1,i},\mathbf{k}'_{1,i},\mathbf{k}_{2,i}} E_0(\mathbf{r}_{0,i})\hat{e}|0_{\mathbf{r}_{2,i}}\rangle\langle 1_{\mathbf{r}_{2,i}}||1_{\mathbf{k}_{2,i}}\rangle\hat{O}'_{\mathbf{k}_{2,i},\mathbf{k}'_{1,i}}\left\langle 1_{\mathbf{k}'_{1,i}}\right||1_{\mathbf{r}_{1,i}}\rangle\langle 1_{\mathbf{r}_{1,i}}||1_{\mathbf{k}_{1,i}}\rangle\hat{O}_{\mathbf{k}_{1,i},\mathbf{k}_{0,i}}\langle 1_{\mathbf{k}_{0,i}}|\\
&= \sum_{\mathbf{k}_{0,i},\mathbf{k}_{1,i},\mathbf{k}'_{1,i},\mathbf{k}_{2,i}} E_0(\mathbf{r}_{0,i})\hat{e}|0_{\mathbf{r}_{2,i}}\rangle h(\mathbf{r}_{2,i},\mathbf{k}_{2,i};\mathbf{r}_{1,i},\mathbf{k}'_{1,i})\langle 1_{\mathbf{r}_{1,i}}||1_{\mathbf{k}_{1,i}}\rangle\hat{O}_{\mathbf{k}_{1,i},\mathbf{k}_{0,i}}\langle 1_{\mathbf{k}_{0,i}}|,
\end{aligned} \tag{S4}$$

$\mathbf{k}_{1,s}$ and $\mathbf{k}_{1,i}$ are the wavevectors of the signal and idler photons after the first $4f$ systems, respectively. Similarly, $\mathbf{k}'_{1,s}$ and $\mathbf{k}'_{1,i}$ are the wavevectors from the object and reference planes to

the second 4f systems in the signal and idler arms, respectively. $\mathbf{k}_{2,s}$ and $\mathbf{k}_{2,i}$ are the wavevectors on the detection plane, respectively. $h(\mathbf{r}_{2,s},\mathbf{k}_{2,s};\mathbf{r}_{1,s},\mathbf{k}'_{1,s}) = \langle 1_{\mathbf{r}_{2,s}}||1_{\mathbf{k}_{2,s}}\rangle \hat{O}'_{\mathbf{k}_{2,s},\mathbf{k}'_{1,s}} \left\langle 1_{\mathbf{k}'_{1,s}}\right||1_{\mathbf{r}_{1,s}}\rangle$ describes the photon propagation from $\mathbf{r}_{1,s}$ to $\mathbf{r}_{2,s}$ through a 4f system. Other h functions can be interpreted accordingly. Defining the phase shift related to $h(\mathbf{r}_{2,s},\mathbf{k}_{2,s};\mathbf{r}_{1,s},\mathbf{k}'_{1,s})$ as $\phi_{s,21}$, we write $h(\mathbf{r}_{2,s},\mathbf{k}_{2,s};\mathbf{r}_{1,s},\mathbf{k}'_{1,s}) = e^{i\phi_{s,21}}$. The phase changes $\phi_{s,10}$, $\phi_{i,21}$, and $\phi_{i,10}$ can be interpreted in a similar way. The SPDC source and the symmetric alignment ensure that paired photons take almost symmetric optical paths; the 4f systems ensure conjugations among the source, object or reference, and detection planes. As a result, the phase changes of the signal and idler photons from the source Fourier plane to the object and reference planes are identical, $\phi_{s,10} = \phi_{i,10} = \phi_{10}$. Similarly, the phase changes from the object and reference planes to the detection plane are identical, $\phi_{s,21} = \phi_{i,21} = \phi_{21}$ (see Discussion). We combine $\hat{E}_s^{(+)}$, $\hat{E}_i^{(+)}$, and $|\xi\rangle$:

$$\begin{aligned}
&\hat{E}_s^{(+)}(\mathbf{r}_{2,s},\mathbf{r}_{1,s},\mathbf{r}_{0,s})\hat{E}_i^{(+)}(\mathbf{r}_{2,i},\mathbf{r}_{1,i},\mathbf{r}_{0,i})|\xi\rangle \\
&= E_0(\mathbf{r}_{0,s})E_0(\mathbf{r}_{0,i}) \sum_{\substack{\mathbf{k}_{0,s},\mathbf{k}_{1,s},\mathbf{k}'_{1,s},\mathbf{k}_{2,s},\\ \mathbf{k}_{0,i},\mathbf{k}_{1,i},\mathbf{k}'_{1,i},\mathbf{k}_{2,i}}} |0_{\mathbf{r}_{2,s}},0_{\mathbf{r}_{2,i}}\rangle A(\mathbf{k}_{0,s}) \times \\
&t_0(\mathbf{r}_{1,s},\mathbf{k}_{1,s})h(\mathbf{r}_{2,s},\mathbf{k}_{2,s};\mathbf{r}_{1,s},\mathbf{k}'_{1,s})h(\mathbf{r}_{1,s},\mathbf{k}_{1,s};\mathbf{r}_{0,s},\mathbf{k}_{0,s})h(\mathbf{r}_{2,i},\mathbf{k}_{2,i};\mathbf{r}_{1,i},\mathbf{k}'_{1,i})h(\mathbf{r}_{1,i},\mathbf{k}_{1,i};\mathbf{r}_{0,i},\mathbf{k}_{0,i}) \\
&= E_0(\mathbf{r}_{0,s})E_0(\mathbf{r}_{0,i}) \sum_{\substack{\mathbf{k}_{0,s},\mathbf{k}_{1,s},\mathbf{k}'_{1,s},\mathbf{k}_{2,s},\\ \mathbf{k}_{0,i},\mathbf{k}_{1,i},\mathbf{k}'_{1,i},\mathbf{k}_{2,i}}} |0_{\mathbf{r}_{2,s}},0_{\mathbf{r}_{2,i}}\rangle A(\mathbf{k}_{0,s})t_0(\mathbf{r}_{1,s},\mathbf{k}_{1,s})e^{j\phi_{s,21}}e^{j\phi_{s,10}}e^{j\phi_{i,21}}e^{j\phi_{i,10}} \\
&= E_0(\mathbf{r}_{0,s})E_0(\mathbf{r}_{0,i}) \sum_{\mathbf{k}_{0,s},\mathbf{k}_{1,s},\mathbf{k}'_{1,s},\mathbf{k}_{2,s}} |0_{\mathbf{r}_{2,s}},0_{\mathbf{r}_{2,i}}\rangle A(\mathbf{k}_{0,s})t_0(\mathbf{r}_{1,s},\mathbf{k}_{1,s})e^{j2\phi_{s,21}}e^{j2\phi_{s,10}} \\
&= E_0(\mathbf{r}_{0,s})E_0(\mathbf{r}_{0,i}) \sum_{\mathbf{k}_{0,s},\mathbf{k}_{1,s},\mathbf{k}'_{1,s},\mathbf{k}_{2,s}} |0_{\mathbf{r}_{2,s}},0_{\mathbf{r}_{2,i}}\rangle \times \\
&\qquad A(\mathbf{k}_{0,s})t_0(\mathbf{r}_{1,s},\mathbf{k}_{1,s})h(\mathbf{r}_{2,s},2\mathbf{k}_{2,s};\mathbf{r}_{1,s},2\mathbf{k}'_{1,s})h(\mathbf{r}_{1,s},2\mathbf{k}_{1,s};\mathbf{r}_{0,s},2\mathbf{k}_{0,s}),
\end{aligned} \tag{S5}$$

$\mathbf{k}_{0,i}, \mathbf{k}_{1,i}, \mathbf{k}'_{1,i}$, and $\mathbf{k}_{2,i}$ are removed because

$$e^{j2\phi_{s,21}}e^{j2\phi_{s,10}} = h(\mathbf{r}_{2,s},2\mathbf{k}_{2,s};\mathbf{r}_{1,s},2\mathbf{k}'_{1,s})h(\mathbf{r}_{1,s},2\mathbf{k}_{1,s};\mathbf{r}_{0,s},2\mathbf{k}_{0,s}), \tag{S6}$$

which is not related to $\mathbf{k}_{0,i}, \mathbf{k}_{1,i}, \mathbf{k}'_{1,i}$, or $\mathbf{k}_{2,i}$. Because the amplitude distribution on the source Fourier plane is symmetric, for an entangled photon pair, $E_0(\mathbf{r}_{0,i}) = E_0(\mathbf{r}_{0,s})$. Substituting Eq. (S5) into Eq. (6) yields

$$G_{\mathrm{QMC}}^{(2)}(\mathbf{r}_{2,s},\mathbf{r}_{1,s},\mathbf{r}_{0,s}) = \left|t_0'(\mathbf{r}_{1,s};\mathbf{r}_{0,s})\right|^2 \left|h\left(\frac{\lambda}{2};\mathbf{r}_{2,s},\mathbf{r}_{1,s}\right)\right|^2 \left|E_0^2(\mathbf{r}_{0,s})h\left(\frac{\lambda}{2};\mathbf{r}_{1,s},\mathbf{r}_{0,s}\right)\right|^2. \quad \text{(S7)}$$

Here, $h\left(\frac{\lambda}{2};\mathbf{r}_{2,s},\mathbf{r}_{1,s}\right) = \sum_{\mathbf{k}_{1,s}',\mathbf{k}_{2,s}} h(\mathbf{r}_{2,s},2\mathbf{k}_{2,s};\mathbf{r}_{1,s},2\mathbf{k}_{1,s}')$ and $h\left(\frac{\lambda}{2};\mathbf{r}_{1,s},\mathbf{r}_{0,s}\right) = \sum_{\mathbf{k}_{0,s},\mathbf{k}_{1,s}} h(\mathbf{r}_{1,s},2\mathbf{k}_{1,s};\mathbf{r}_{0,s},2\mathbf{k}_{0,s})$ are the PSFs from $\mathbf{r}_{1,s}$ to $\mathbf{r}_{2,s}$ and from $\mathbf{r}_{0,s}$ to $\mathbf{r}_{1,s}$ with light of $\lambda/2$ wavelength, respectively, where λ is the wavelength of the SPDC photons. $t_0'(\mathbf{r}_{1,s};\mathbf{r}_{0,s})$ is given by

$$t_0'(\mathbf{r}_{1,s};\mathbf{r}_{0,s}) = \frac{\sum_{\mathbf{k}_{0,s},\mathbf{k}_{1,s}} A(\mathbf{k}_{0,s})t_0(\mathbf{r}_{1,s},\mathbf{k}_{1,s})h(\mathbf{r}_{1,s},2\mathbf{k}_{1,s};\mathbf{r}_{0,s},2\mathbf{k}_{0,s})}{\sum_{\mathbf{k}_{0,s},\mathbf{k}_{1,s}} h(\mathbf{r}_{1,s},2\mathbf{k}_{1,s};\mathbf{r}_{0,s},2\mathbf{k}_{0,s})}. \quad \text{(S8)}$$

For simplicity, we then set $A(\mathbf{k}_{0,s}) = A_0$ within the summation range of $\mathbf{k}_{0,s}$. If $t_0(\mathbf{r}_{1,s},\mathbf{k}_{1,s})$ is insensitive to $\mathbf{k}_{1,s}$, $t_0'(\mathbf{r}_{1,s};\mathbf{r}_{0,s})$ becomes approximately unrelated to $\mathbf{r}_{0,s}$ and is reduced to $A_0 t(\mathbf{r}_{1,s})$, which is the amplitude transmission coefficient of the object that only depends on the position $\mathbf{r}_{1,s}$. For a wide-field microscope, we then need to integrate Eq. (S7) over all the possible source positions $\mathbf{r}_{0,s}$. Letting $\Gamma_{\mathrm{QMC}}\left(\frac{\lambda}{2};\mathbf{r}_{1,s}\right) = |A_0|^2 \int_S \left|E_0^2(\mathbf{r}_{0,s})h\left(\frac{\lambda}{2};\mathbf{r}_{1,s},\mathbf{r}_{0,s}\right)\right|^2 d\mathbf{r}_{0,s}$, we have

$$G_{\mathrm{QMC}}^{(2)}(\mathbf{r}_{2,s},\mathbf{r}_{1,s}) = \left|t(\mathbf{r}_{1,s})\right|^2 \Gamma_{\mathrm{QMC}}\left(\frac{\lambda}{2};\mathbf{r}_{1,s}\right)\left|h\left(\frac{\lambda}{2};\mathbf{r}_{2,s},\mathbf{r}_{1,s}\right)\right|^2. \quad \text{(S9)}$$

$\Gamma_{\mathrm{QMC}}\left(\frac{\lambda}{2};\mathbf{r}_{1,s}\right)$ is the distribution of squared intensity on the object plane with wavelength $\lambda/2$.

The intensity of the signal photons (classical imaging) is given by the first-order correlation function:

$$G_{\mathrm{CI}}^{(1)} = \left|\left\langle 0\left|\hat{E}_s^{(+)}\right|\xi_s\right\rangle\right|^2, \quad \text{(S10)}$$

where $|\xi_s\rangle$ denotes the wavefunction of the signal photon emitted from $\boldsymbol{r}_{0,s}$:

$$|\xi_s\rangle = \sum_{\mathbf{k}_{0,s}} A_s(\mathbf{k}_{0,s})e^{-j\mathbf{k}_{0,s}\cdot\mathbf{r}_{0,s}}\left|1_{\mathbf{k}_{0,s}}\right\rangle. \quad \text{(S11)}$$

where $A_s(\mathbf{k}_{0,s})$ denotes the probability amplitude of the state $\left|1_{\mathbf{k}_{0,s}}\right\rangle$. Substituting Eq. (S3) and Eq. (S11) to Eq. (S10), we obtain

$$\hat{E}_s^{(+)}\left(\mathbf{r}_{2,s},\mathbf{r}_{1,s},\mathbf{r}_{0,s}\right)|\xi_s\rangle = E_0\left(\mathbf{r}_{0,s}\right)\hat{e}\sum_{\mathbf{k}_{0,s},\mathbf{k}_{1,s},\mathbf{k}'_{1,s},\mathbf{k}_{2,s}}\left|0_{\mathbf{r}_{2,s}}\right\rangle A_s\left(\mathbf{k}_{0,s}\right)t_0\left(\mathbf{r}_{1,s},\mathbf{k}_{1,s}\right)e^{j\phi_{s,21}}e^{j\phi_{s,10}}$$

$$= E_0\left(\mathbf{r}_{0,s}\right)\hat{e}\sum_{\mathbf{k}_{0,s},\mathbf{k}_{1,s},\mathbf{k}'_{1,s},\mathbf{k}_{2,s}}\left|0_{\mathbf{r}_{2,s}}\right\rangle A_s\left(\mathbf{k}_{0,s}\right)t_0\left(\mathbf{r}_{1,s},\mathbf{k}_{1,s}\right)h\left(\mathbf{r}_{2,s},\mathbf{k}_{2,s};\mathbf{r}_{1,s},\mathbf{k}'_{1,s}\right)h\left(\mathbf{r}_{1,s},\mathbf{k}_{1,s};\mathbf{r}_{0,s},\mathbf{k}_{0,s}\right). \quad \text{(S12)}$$

Thus, with the same assumption in Eq. (S9) that $A_s\left(\mathbf{k}_{0,s}\right) = A_{s0}$, and $t_0\left(\mathbf{r}_{1,s},\mathbf{k}_{1,s}\right)$ is not sensitive to $\mathbf{k}_{1,s}$, we have

$$G_{\mathrm{CI}}^{(1)}\left(\mathbf{r}_{2,s},\mathbf{r}_{1,s},\mathbf{r}_{0,s}\right) = \left|A_{s0}t\left(\mathbf{r}_{1,s}\right)\right|^2\left|h\left(\lambda;\mathbf{r}_{2,s},\mathbf{r}_{1,s}\right)\right|^2\left|E_0\left(\mathbf{r}_{0,s}\right)h\left(\lambda;\mathbf{r}_{1,s},\boldsymbol{r}_{0,s}\right)\right|^2. \quad \text{(S13)}$$

Here, $h\left(\lambda;\mathbf{r}_{2,s},\mathbf{r}_{1,s}\right)$ and $h\left(\lambda;\mathbf{r}_{1,s},\mathbf{r}_{0,s}\right)$ demonstrate the PSFs in the signal arm. We then integrate Eq. (S13) over all the possible source positions $\mathbf{r}_{0,s}$, giving:

$$G_{\mathrm{CI}}^{(1)}\left(\mathbf{r}_{2,s},\mathbf{r}_{1,s}\right) = \left|t\left(\mathbf{r}_{1,s}\right)\right|^2\gamma_{\mathrm{CI}}\left(\lambda;\mathbf{r}_{1,s}\right)\left|h\left(\lambda;\mathbf{r}_{1,s},\mathbf{r}_{2,s}\right)\right|^2. \quad \text{(S14)}$$

where $\gamma_{\mathrm{CI}}\left(\lambda;\mathbf{r}_{1,s}\right) = |A_{s0}|^2\int_S\left|E_0\left(\mathbf{r}_{0,s}\right)h\left(\lambda;\mathbf{r}_{1,s},\mathbf{r}_{0,s}\right)\right|^2 d\mathbf{r}_{0,s}$, which is the intensity distribution of the wide-field illumination with wavelength λ. If the amplitude distribution of the electric field on the source Fourier plane is uniform, i.e., $E_0\left(\mathbf{r}_{0,s}\right) = E_0(0)$,

$$\Gamma_{\mathrm{QMC}}\left(\frac{\lambda}{2};\mathbf{r}_{1,s}\right) = |A_0|^2|E_0(0)|^4\int_S\left|h\left(\frac{\lambda}{2};\mathbf{r}_{1,s},\mathbf{r}_{0,s}\right)\right|^2 d\mathbf{r}_{0,s} \quad \text{(S15)}$$

is proportional to

$$\gamma_{\mathrm{CI}}\left(\frac{\lambda}{2};\mathbf{r}_{1,s}\right) = |A_{s0}|^2|E_0(0)|^2\int_S\left|h\left(\frac{\lambda}{2};\mathbf{r}_{1,s},\mathbf{r}_{0,s}\right)\right|^2 d\mathbf{r}_{0,s}. \quad \text{(S16)}$$

We replace the 3D vectors $\mathbf{r}_{1,s}$ by a 2D vector $\boldsymbol{\rho}$ on the object plane. Denoting the imaging magnification from $\mathbf{r}_{1,s}$ to $\mathbf{r}_{2,s}$ as M, the QMC and the classical images become

$$G_{\mathrm{QMC}}^{(2)}(\boldsymbol{\rho}) = |t(\boldsymbol{\rho})|^2\Gamma_{\mathrm{QMC}}\left(\frac{\lambda}{2};\boldsymbol{\rho}\right)\left|h\left(\frac{\lambda}{2};\boldsymbol{\rho},M\boldsymbol{\rho}\right)\right|^2, \quad \text{(S17)}$$

$$G_{\mathrm{CI}}^{(1)}(\boldsymbol{\rho}) = |t(\boldsymbol{\rho})|^2\gamma_{\mathrm{CI}}(\lambda;\boldsymbol{\rho})|h(\lambda;\boldsymbol{\rho},M\boldsymbol{\rho})|^2, \quad \text{(S18)}$$

where $t(\boldsymbol{\rho})$ is the amplitude transmission coefficient of the object, and $\boldsymbol{\rho}$ is the 2D coordinates on the object plane. $h(\lambda;\boldsymbol{\rho},M\boldsymbol{\rho})$ denotes the point spread function (PSF) from $\boldsymbol{\rho}$ on the object plane (P_{obj}) to $M\boldsymbol{\rho}$ on the detection plane (P_{det}) for the light with a wavelength of λ. The other PSFs can be interpreted accordingly.

If $M = 1$, Eqs. (S17) and (S18) can be simplified as

$$G_{\mathrm{QMC}}^{(2)}(\boldsymbol{\rho}) = |t(\boldsymbol{\rho})|^2 \Gamma_{\mathrm{QMC}}\left(\frac{\lambda}{2}; \boldsymbol{\rho}\right) \left| h\left(\frac{\lambda}{2}\right) \right|^2 , \tag{S19}$$

$$G_{\mathrm{CI}}^{(1)}(\boldsymbol{\rho}) = |t(\boldsymbol{\rho})|^2 \gamma_{\mathrm{CI}}(\lambda; \boldsymbol{\rho}) |h(\lambda)|^2 . \tag{S20}$$

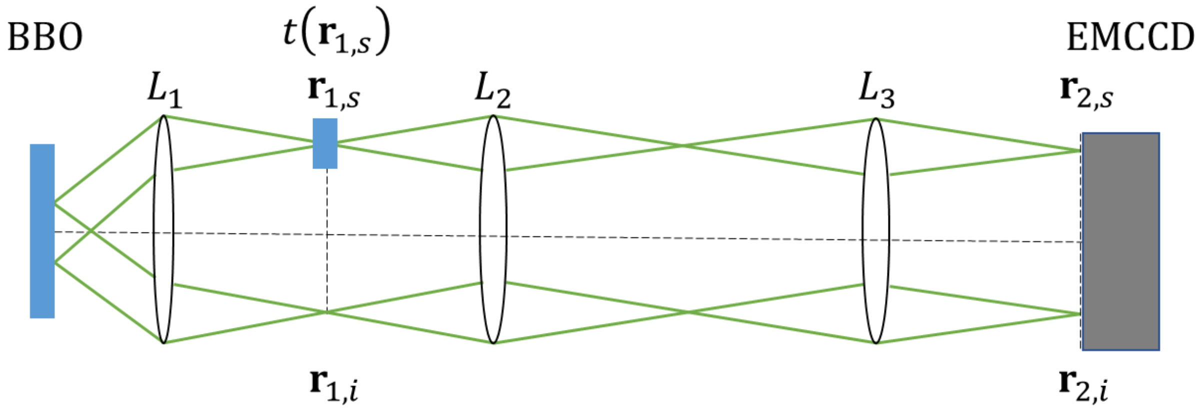

Supplementary Fig. 1 Simplified schematic of previous wide-field quantum imaging setups.

A simplified schematic of previous wide-field quantum imaging[23,24]. BBO, β-barium borate crystals. EMCCD, electron multiplying charge-coupled device camera. $\mathbf{r}_{1,s}$ and $\mathbf{r}_{2,s}$ are the coordinates of the object plane and the detection plane in the signal arm, and $\mathbf{r}_{1,i}$, and $\mathbf{r}_{2,i}$ are the coordinates of the reference plane and the detection plane in the idler arm. t is the amplitude transmission coefficient of the object. L_1, L_2, L_3, lenses.

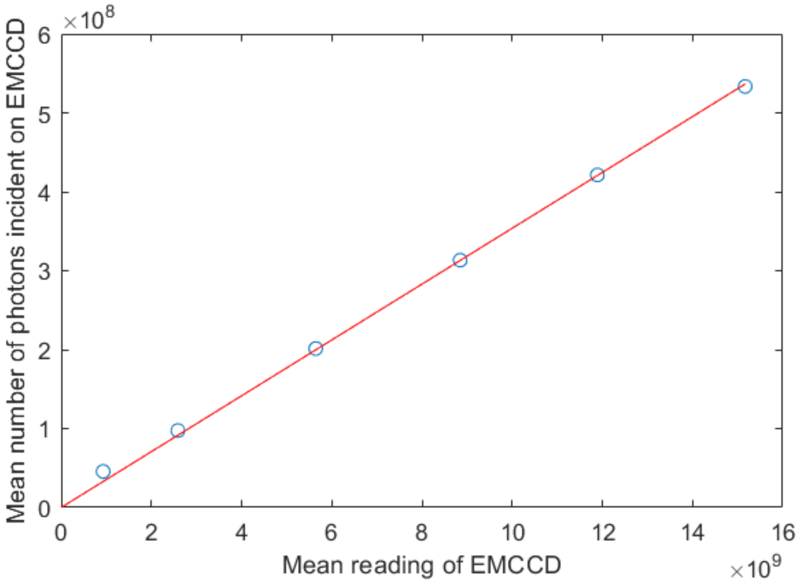

Supplementary Fig. 2 Calibration for the EMCCD.

Experimental calibration of the relation between the mean number of photons incident on the EMCCD and the mean reading of the EMCCD per frame with a ×1000 gain. In the absence of signal photons, the EMCCD reading indicates an averaged background noise of 467 (see Supplementary Fig. 3). The red curve is a linear fit to the calibration data. The slope of the curve is 0.037. In the case that the mean reading of the EMCCD per pixel per frame is 13, the corresponding mean number of photons incident on the EMCCD is 0.49 per pixel per frame.

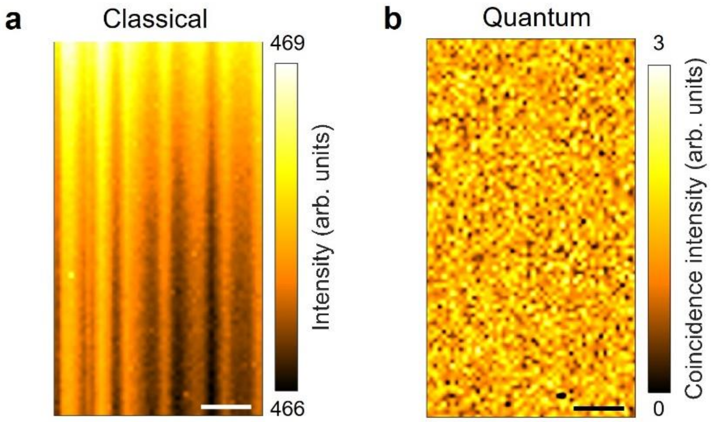

Supplementary Fig. 3 Covariance of noise in the absence of signal.

Classical image (**a**) and the corresponding QMC image (**b**) of the noise. Both images are averaged over 10^5 frames. The mean of the noise intensity in **a** is ~467. The coincidence intensity of noise in **b**, labeled as coincidence intensity, has a mean of ~0. Scale bars, 20 μm. Arb. units, arbitrary units.

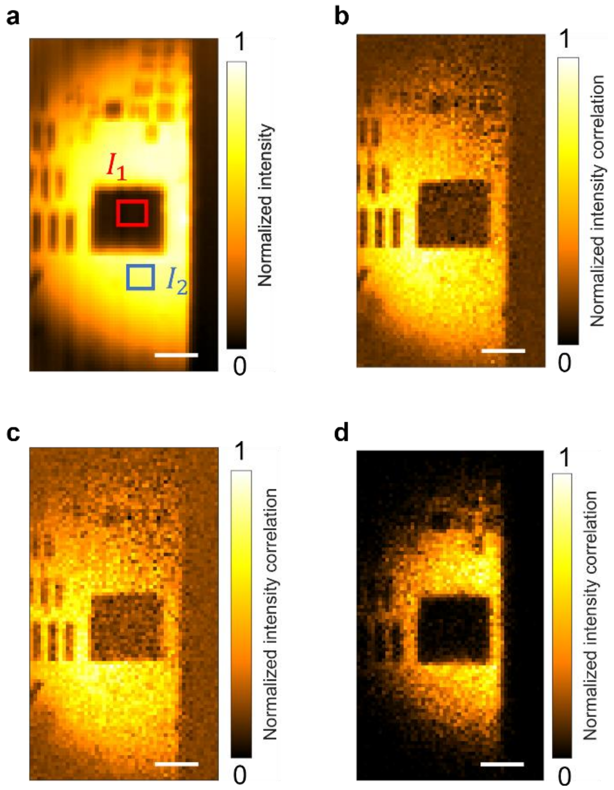

Supplementary Fig. 4 CNR estimation for Fig. 2b.

a, Classical image of a USAF resolution target. The red and blue rectangular areas correspond to the object of interest (I_1) and the background (I_2). Both rectangular areas were positioned differently 10 times to estimate the standard errors of the CNRs. The CNR is defined in Methods. **b–d**, QMC images computed using the covariance algorithm (**b**), the algorithm in Refs. [23,32] (**c**), and the algorithm in Ref. [24] (**d**). All images are averaged over 2×10^6 frames. The CNRs are 8.58 ± 0.53 (**b**), 5.55 ± 0.23 (**c**), and 3.30 ± 0.29 (**d**), respectively. When estimating the CNRs, we did not consider the green dashed area in **b**, which was degraded due to imperfect alignment.

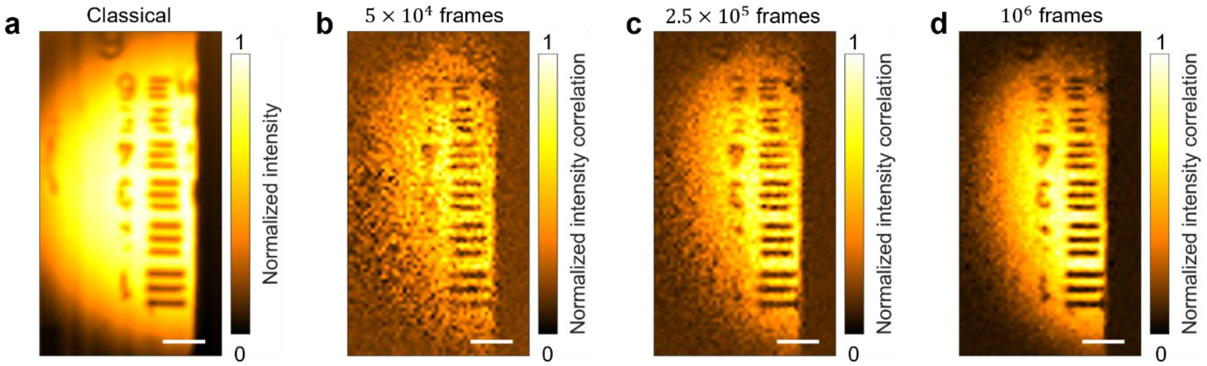

Supplementary Fig. 5 QMC images with different numbers of frames.

(**a**), Classical image of group 7 of a USAF 1951 resolution target. QMC images of the same FOV using 5×10^4 (**b**), 2.5×10^5 (**c**), and 10^6 (**d**) frames. The CNR increases from 1.9 to 4.1 and then to 7.1. Scale bars, 20 µm.

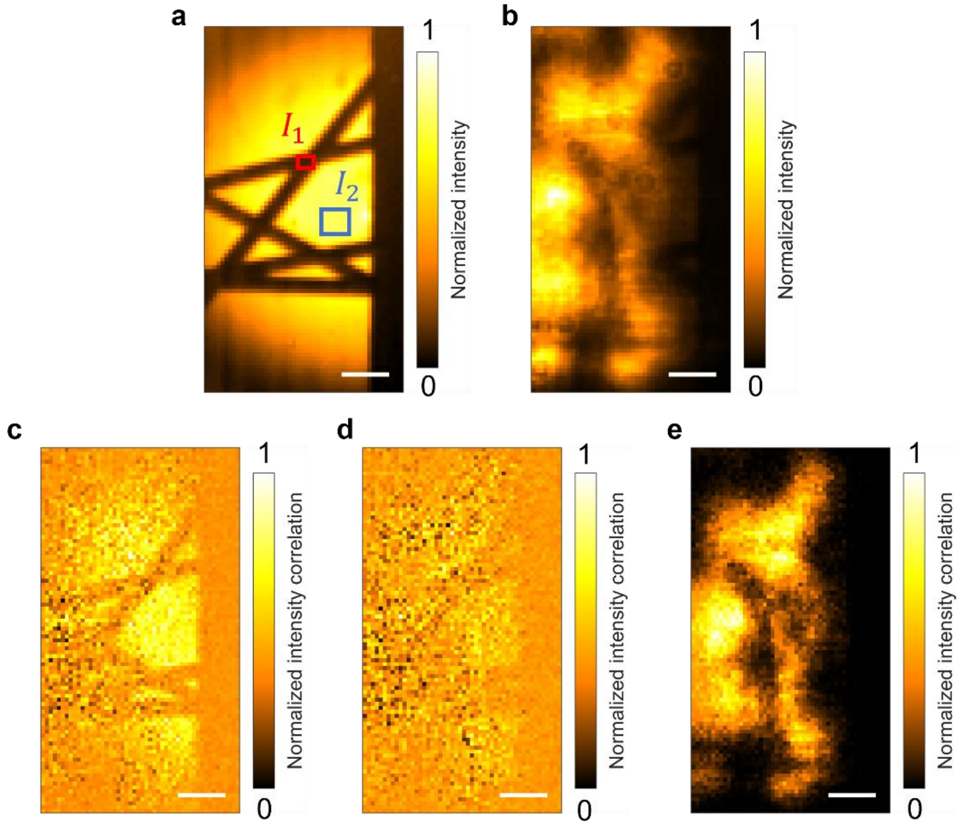

Supplementary Fig. 6 Estimation of stray light resistance for Fig. 2c.

a, Classical image of carbon fibers. The red and blue rectangular areas correspond to the object of interest (I_1) and the background (I_2). Both rectangular areas were positioned differently 10 times to estimate the standard errors of the CNRs. The CNR is defined in the Methods. **b**, Classical image contaminated by stray light 12 times stronger than the classical signal. **c**–**e**, QMC images computed using the covariance algorithm (**c**), the algorithm in Refs. [23,32] (**d**), and the algorithm in Ref. [24] (**e**). All images are averaged over 10^5 frames. The CNRs are 0.94 ± 0.09 (**b**), 2.80 ± 0.33 (**c**), 0.82 ± 0.07 (**d**), and 0.82 ± 0.05 (**e**), respectively.

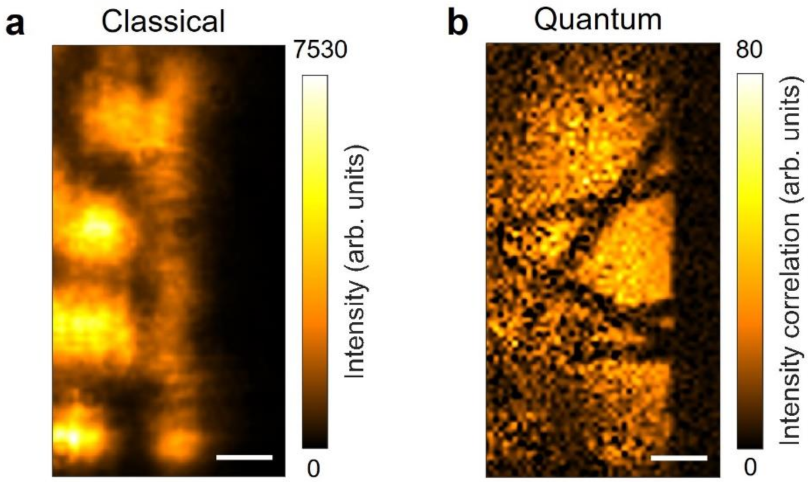

Supplementary Fig. 7 Resistance to strong stray light.

Classical image (**a**) and the corresponding QMC image (**b**) with stray light 155 times stronger than the classical signal. Both images are averaged over 2×10^6 frames. Scale bars, 20 μm. The CNRs of the classical imaging and QMC are 0.11 ± 0.62 and 3.56 ± 0.50 (n = 10). The mean EMCCD reading of the stray light is 2046, corresponding to 76 photons incident on the EMCCD per pixel per frame, which is ~155 times greater than the mean number of the signal photons (0.49 per pixel per frame). Arb. units, arbitrary units.

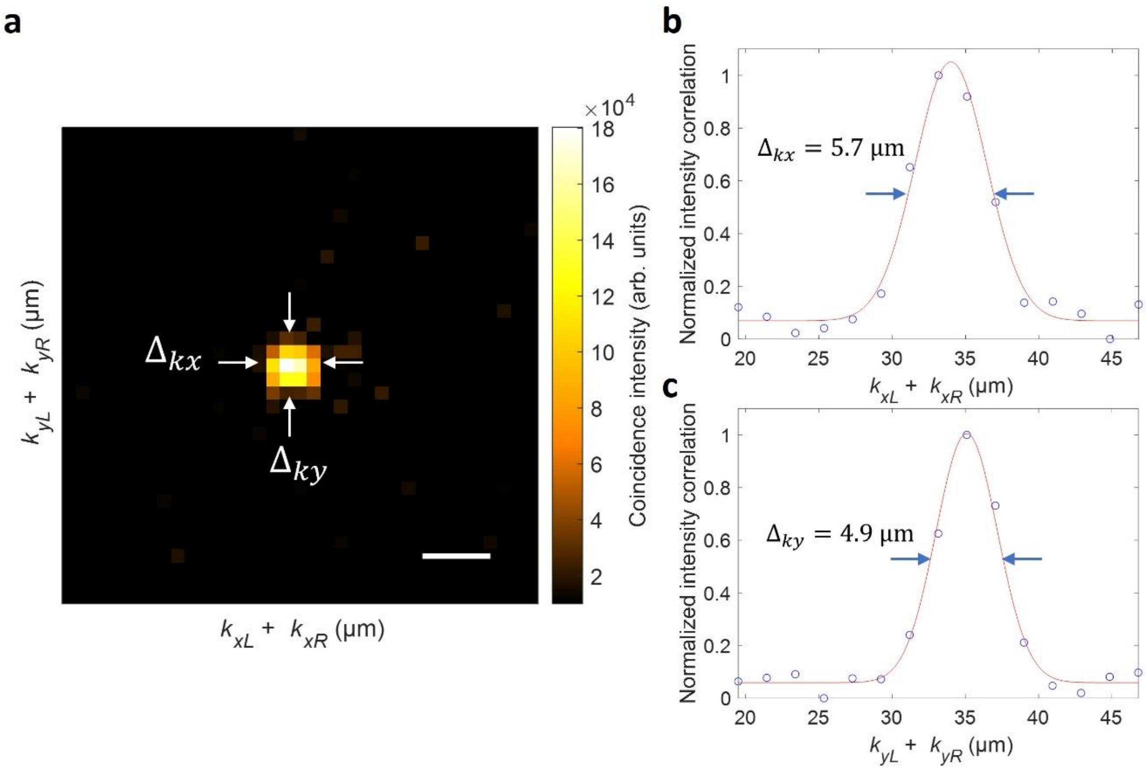

Supplementary Fig. 8 Momentum correlation width.

a, Distribution of coincidence intensity represented in the sum-coordinate axis $k_{xL} + k_{xR}$ and $k_{yL} + k_{yR}$. Scale bar, 10 μm. The source Fourier plane in this experiment is the Fourier plane of the BBO crystal, which is also related to the detection plane, so the coordinate corresponds to a unit of length. Arb. units, arbitrary units. **b**, Normalized intensity correlation along the $\mathbf{k}_x$ direction. The momentum correlation width is 5.7 μm. **c**, Normalized intensity correlation along the $\mathbf{k}_y$ direction. The momentum correlation width is 4.9 μm. The curves in **b** and **c** are Gaussian fits.

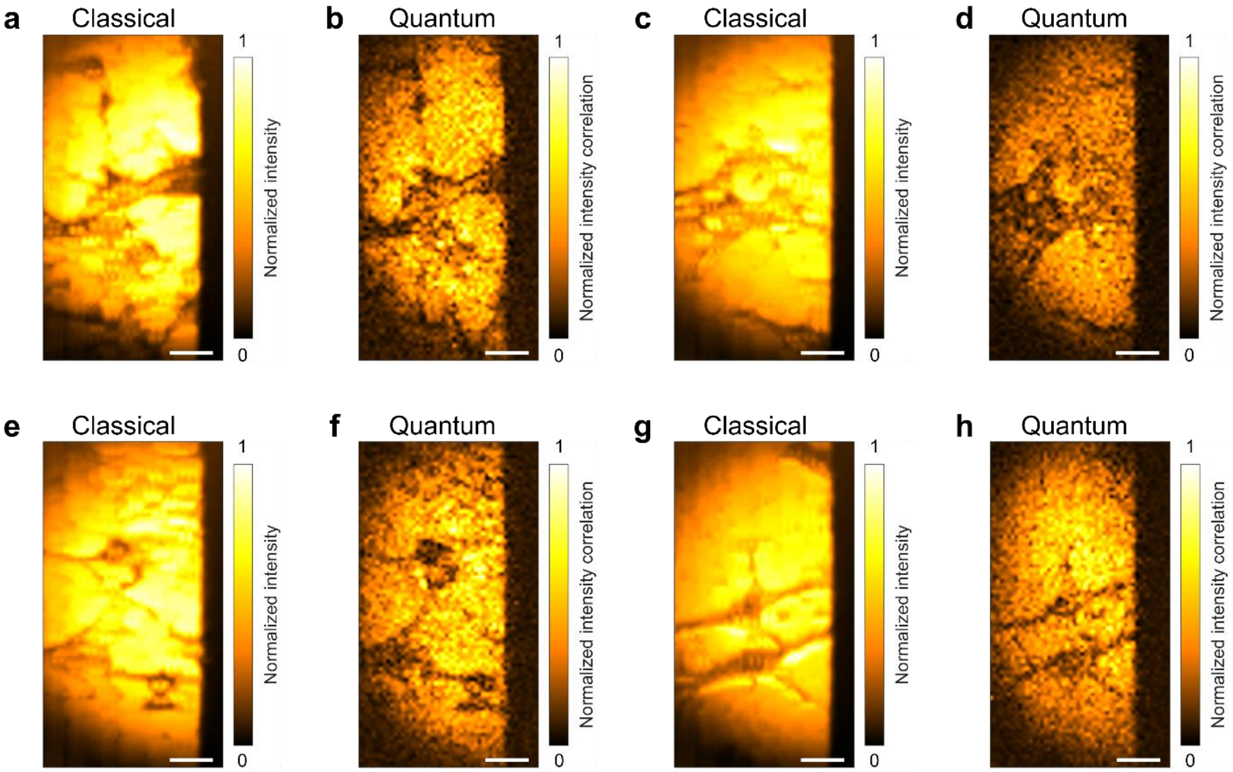

Supplementary Fig. 9 QMC images of different cancer cells acquired with 10^5 frames.

a, **c**, **e**, **g**, Classical images averaged over 10^5 frames of different HeLa cells. **b**, **d**, **f**, **h**, Corresponding QMC images using 10^5 frames. Scale bars, 20 μm.
