# Supplementary material for: Quantum microscopy of cells at the Heisenberg limit
Source: Nat Commun. 2023 Apr 28;14:2441. doi: 10.1038/s41467-023-38191-4 (PMC10147633; doi:10.1038/s41467-023-38191-4)
Supplement: Supplementary file 2 — Reporting Summary [file 41467_2023_38191_MOESM2_ESM.pdf]

nature portfolio

Corresponding author(s): Lihong V. Wang

Last updated by author(s): Mar 20, 2023

Reporting Summary

Nature Portfolio wishes to improve the reproducibility of the work that we publish. This form provides structure for consistency and transparency in reporting. For further information on Nature Portfolio policies, see our Editorial Policies and the Editorial Policy Checklist.

Statistics

For all statistical analyses, confirm that the following items are present in the figure legend, table legend, main text, or Methods section.

n/a	Confirmed	
☐	☒	The exact sample size (*n*) for each experimental group/condition, given as a discrete number and unit of measurement
☐	☒	A statement on whether measurements were taken from distinct samples or whether the same sample was measured repeatedly
☒	☐	The statistical test(s) used AND whether they are one- or two-sided<br>*Only common tests should be described solely by name; describe more complex techniques in the Methods section.*
☒	☐	A description of all covariates tested
☒	☐	A description of any assumptions or corrections, such as tests of normality and adjustment for multiple comparisons
☐	☒	A full description of the statistical parameters including central tendency (e.g. means) or other basic estimates (e.g. regression coefficient) AND variation (e.g. standard deviation) or associated estimates of uncertainty (e.g. confidence intervals)
☒	☐	For null hypothesis testing, the test statistic (e.g. *F*, *t*, *r*) with confidence intervals, effect sizes, degrees of freedom and *P* value noted<br>*Give P values as exact values whenever suitable.*
☒	☐	For Bayesian analysis, information on the choice of priors and Markov chain Monte Carlo settings
☒	☐	For hierarchical and complex designs, identification of the appropriate level for tests and full reporting of outcomes
☒	☐	Estimates of effect sizes (e.g. Cohen's *d*, Pearson's *r*), indicating how they were calculated

Our web collection on statistics for biologists contains articles on many of the points above.

Software and code

Policy information about availability of computer code

Data collection	LabVIEW version 18.0.1f4 (National Instruments, Corp.), Andor SOLIS 2017 version 4.30.30034.0
Data analysis	MATLAB version R2021a

For manuscripts utilizing custom algorithms or software that are central to the research but not yet described in published literature, software must be made available to editors and reviewers. We strongly encourage code deposition in a community repository (e.g. GitHub). See the Nature Portfolio guidelines for submitting code & software for further information.

Data

Policy information about availability of data

All manuscripts must include a data availability statement. This statement should provide the following information, where applicable:

- Accession codes, unique identifiers, or web links for publicly available datasets
- A description of any restrictions on data availability
- For clinical datasets or third party data, please ensure that the statement adheres to our policy

All data are available within the Article and Supplementary Information, or available from the corresponding author upon reasonable request. Imaging data of cells and the code for the covariance algorithm are available in Github online: http://github.com/ZheHE2022/Quantum-Microscopy-of-Cells-at-the-Heisenberg-Limit.

Human research participants

Policy information about studies involving human research participants and Sex and Gender in Research.

Reporting on sex and gender	none
Population characteristics	none
Recruitment	none
Ethics oversight	none

Note that full information on the approval of the study protocol must also be provided in the manuscript.

Field-specific reporting

Please select the one below that is the best fit for your research. If you are not sure, read the appropriate sections before making your selection.

- [x] Life sciences
- [ ] Behavioural & social sciences
- [ ] Ecological, evolutionary & environmental sciences

For a reference copy of the document with all sections, see nature.com/documents/nr-reporting-summary-flat.pdf

Life sciences study design

All studies must disclose on these points even when the disclosure is negative.

Sample size	One batch Hela cells culture was used in the study. No statistical method was used to predetermine sample size. We determined sample sizes based on our preliminary studies and on the criteria in the field to experimentally demonstrate the imaging system.
Data exclusions	More cell images were not shown in this work. We exhibited some typical images of cells that shows the advantages of our reported method.
Replication	All experiments except for that shown in Fig. 4 were replicated at least twice. All attempts to replication were successful. Cell imaging in Fig. 4 with 2×10^6 frames was not replicated because we have repeated the measurement under the same condition with 10^5 frames (Supplementary Fig. 9). Besides, we have repeated experiments on other samples under the same condition with 2×10^6 frames.
Randomization	Randomization was not relevant to this study because we only use samples to demonstrate the advantages of our reported method and are not comparing between different samples.
Blinding	Blinding was not applicable during experiments and outcome assessment because we do not compare between samples or populations. The samples are only used to demonstrate the imaging system.

Reporting for specific materials, systems and methods

We require information from authors about some types of materials, experimental systems and methods used in many studies. Here, indicate whether each material, system or method listed is relevant to your study. If you are not sure if a list item applies to your research, read the appropriate section before selecting a response.

Materials & experimental systems

n/a	Involved in the study	
x		Antibodies
	x	Eukaryotic cell lines
x		Palaeontology and archaeology
x		Animals and other organisms
x		Clinical data
x		Dual use research of concern

Methods

n/a	Involved in the study	
x		ChIP-seq
x		Flow cytometry
x		MRI-based neuroimaging

Eukaryotic cell lines

Policy information about cell lines and Sex and Gender in Research

Cell line source(s)	HeLa immortalized cell line from American Type Culture Collection (ATCC)
Authentication	HeLa cell line were obtained from ATCC and were not additionally authenticated.
Mycoplasma contamination	HeLa cell line were obtained from ATCC and were not additionally tested for mycoplasma.
Commonly misidentified lines (See ICLAC register)	No commonly misidentified cell lines were used.
